# Quality of life assessment in patients with negative symptoms

**DOI:** 10.1192/j.eurpsy.2021.1427

**Published:** 2021-08-13

**Authors:** B. Norbutaitė, V. Adomaitienė, D. Leskauskas, K. Butkutė-Šliuožienė, J. Montvidas

**Affiliations:** 1 Department Of Psychiatry, Lithuanian University of health sciences, Kaunas, Lithuania; 2 Psychiatry, Lithuanian University of Health Sciences, Kaunas, Lithuania

**Keywords:** negative symptoms, schizophrénia, SNS scale

## Abstract

**Introduction:**

One or more negative symptoms are present in 57,6% of patients with schizophrenia spectrum disorder [Bobes et al, 2010]. These symptoms are responsible for impaired social functioning and have impact on the quality of life. There are no epidemiological studies that analyse the prevalence of negative symptoms and their impact on life quality in Lithuania.

**Objectives:**

To evaluate the impact of negative symptoms on quality of life in patients with schizophrenia spectrum disorder.

**Methods:**

Participants were 48 adults with schizophrenia (n=36) or schizoaffective disorders (n=12). All participants provided informed consent. All participants were administered a sociodemographic data form, Brief Psychiatric Rating Scale (BPRS), Mini-International Neuropsychiatric Interview (MINI). Negative symptoms were assessed by the Self-evaluation of Negative Symptoms (SNS). The Short-Form Health Survey (SF-36) was used to measure health-related quality of life.

**Results:**

The results of SF-36 scales significantly correlated with SNS subscales. All SNS subscales correlated with general health result, vitality, social functioning and emotional well-being as well as in overal quality of life. Signifficant correlations were observed between the total scores of SNS and physical activity (r=-0,404, p=0,004), general health (r=-0,626, p<0,001), vitality (r=-0,683, p=0,004), social functioning (r=-0,53, p<0,001), role limitations (r=0,354, p=0,014), emotional well-being (r=-0,662, p<0,001) in SF-36 scales.

**Conclusions:**

Negative symptoms of schizophrenia such as social withdrawal, diminished emotional range, alogia, avolition and anhedonia are associated with impaired quality of life. We found a strong relation between negative symptoms and quality of life, however further studies can support this point of view.

